# The value of whole lesion ADC histogram profiling to differentiate between morphologically indistinguishable ring enhancing lesions–comparison of glioblastomas and brain abscesses

**DOI:** 10.18632/oncotarget.24454

**Published:** 2018-04-06

**Authors:** Diana Horvath-Rizea, Alexey Surov, Karl-Titus Hoffmann, Nikita Garnov, Cathrin Vörkel, Patricia Kohlhof-Meinecke, Oliver Ganslandt, Hansjörg Bäzner, Georg Alexander Gihr, Marcell Kalman, Elina Henkes, Hans Henkes, Stefan Schob

**Affiliations:** ^1^ Clinic for Neuroradiology, Katharinenhospital Stuttgart, Stuttgart, Germany; ^2^ Clinic for Diagnostic and Interventional Radiology, University Hospital Leipzig, Leipzig, Germany; ^3^ Department for Neuroradiology, University Hospital Leipzig, Leipzig, Germany; ^4^ Eichamt Leipzig, Leipzig, Germany; ^5^ Department for Pathology, Katharinenhospital Stuttgart, Stuttgart, Germany; ^6^ Clinic for Neurosurgery, Katharinenhospital Stuttgart, Stuttgart, Germany; ^7^ Clinic for Neurology, Katherinenhospital Stuttgart, Stuttgart, Germany

**Keywords:** ring enhancing lesion, glioblastoma, brain abscess, diffusion weighted imaging, histogram analysis

## Abstract

**Background:**

Morphologically similar appearing ring enhancing lesions in the brain parenchyma can be caused by a number of distinct pathologies, however, they consistently represent life-threatening conditions. The two most frequently encountered diseases manifesting as such are glioblastoma multiforme (GBM) and brain abscess (BA), each requiring disparate therapeutical approaches. As a result of their morphological resemblance, essential treatment might be significantly delayed or even ommited, in case results of conventional imaging remain inconclusive. Therefore, our study aimed to investigate, whether ADC histogram profiling reliably can distinguish between both entities, thus enhancing the differential diagnostic process and preventing treatment failure in this highly critical context.

**Methods:**

103 patients (51 BA, 52 GBM) with histopathologically confirmed diagnosis were enrolled. Pretreatment diffusion weighted imaging (DWI) was obtained in a 1.5T system using b values of 0, 500, and 1000 s/mm^2^. Whole lesion ADC volumes were analyzed using a histogram-based approach. Statistical analysis was performed using SPSS version 23.

**Results:**

All investigated parameters were statistically different in comparison of both groups. Most importantly, ADCp10 was able to differentiate reliably between BA and GBM with excellent accuracy (0.948) using a cutpoint value of 70 × 10^−5^ mm^2^ × s^−1^.

**Conclusions:**

ADC whole lesion histogram profiling provides a valuable tool to differentiate between morphologically indistinguishable mass lesions. Among the investigated parameters, the 10th percentile of the ADC volume distinguished best between GBM and BA.

## INTRODUCTION

Ring enhancing lesions (REL) within the brain parenchyma in most cases represent a serious, potentially life-threatening disease [1–9]. Although sharing a similar appearance on cross sectional imaging, the causative pathologies are of either neoplastic, infectious or inflammatory-demyelinating nature [10]. Early and accurate differentiation of REL in the neuroradiological routine is of great importance, since the distinct underlying conditions require immediate therapy, with respective treatment options ranging from conservative medical treatment to major brain surgery. Attribution of REL to an incorrect underlying pathology most certainly delays essential treatment and possibly leads to unnecessary extensive brain surgery of a non-surgical lesion, resulting in significant therapy-related morbidity and mortality [11]. In the clinical setting it is most frequently of great importance to distinguish glioblastoma multiforme (GBM) from BA (BA). Both entities present strikingly similar in neuroimaging, but prognosis and management is very distinct. GBM requires en-bloc resection of the whole contrast enhancing portion plus a significant fraction of the adjacent Flair-hyperintense tissue in order to achieve the best possible outcome, which overall remains poor [12]. In contrary, BA necessitates a much less invasive approach - stereotactic aspiration and antimicrobial therapy - which results in complete recovery without permanent neurological sequelae if performed appropriately [13].

The best modality to diagnose and specify REL is cranial magnetic resonance imaging (MRI) [11, 14]. Conventional MRI, including T1 weighted, T2 weighted and contrast enhanced imaging, is frequently used to identify the exact anatomic location of the lesion in question and classify it according its mass effect, perifocal edema, necrosis, hemorrhage and blood-brain barrier (BBB) disruption [14, 15]. Despite the variety of information obtained by conventional MRI, definite differentiation between GBM and brain abscess remains impossible in a number of scenarios and a more functional imaging concept is necessary to increase distinguishability of both entities [16, 17]. Diffusion weighted imaging, measuring the motion of hydrogen nuclei in biological tissues on a microscopical scale [18], is able to reflect histopathological properties of malignant tumors *in vivo* [19–21], and therefore complements the role of conventional MRI in the differentiation of morphologically similarly appearing mass lesions [17]. In general, mean water diffusivity is decreased within abscesses [16] and higher in cystic tumor compartments [22]. However, in up to 21% of untreated abscesses high diffusivity values, as comparably measurable in necrotic or cystic tumors, have been reported [23, 24]. Vice versa, necrotic GBMs can exhibit low diffusivity values, and hence, may be misdiagnosed as abscesses [25, 26]. Thus, DWI, using the standardly applied method of 2-dimensional region of interest (ROI) measurements on ADC maps certainly is superior to conventional MRI in this regard, but still remains inconclusive in a considerable number of cases. An advanced method to quantitatively analyze ADC data of a ring enhancing, possibly malignant lesion is to issue a histogram of the whole three-dimensional ADC volume corresponding to the contrast enhancing portion of the lesion in question. Histogram analysis derived parameters, e.g. skewness, entropy, kurtosis and percentils, describe the diffusion profile of a complex lesion in biological tissues more explicitly than the common concept of ADCmean, ADCmin and ADCmax [27–29]. Hence, histogram analysis of a complete tumor ADC volume more precisely reflects its histopathological features, like cellularity and tumor heterogeneity, and furthermore can predict prognostically important facets of tumor biology like proliferative activity and dedifferentiation [28, 29].

To the best of our knowledge, no study investigated the potential of ADC volume histogram profiling for differentiation of REL so far. Therefore, the aim of this study was to elucidate, whether histogram profiling of whole lesion ADC volumes is a useful tool to distinguish between morphologically resembling cases of GBM and BA, thus aiding the neurosurgeon in identification of the most suitable treatment algorithm.

## RESULTS

### Diffusion weighted imaging

**Figure 1:**
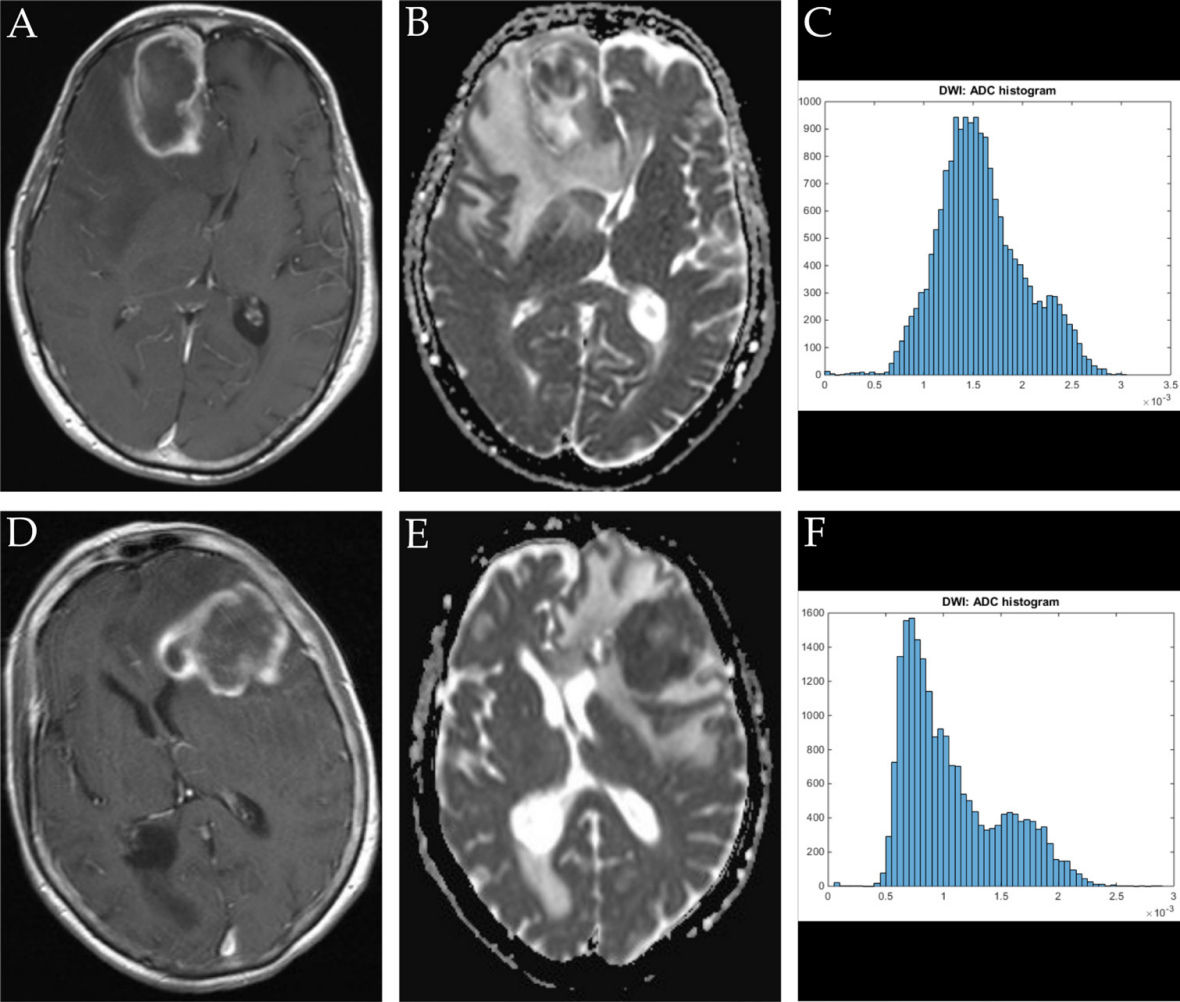
Imaging findings and corresponding ADC-histograms of morphologically resembling frontal lobe manifestations of GBM and brain abscess The upper row shows a representative axial T1 weighted post contrast image (**A**), the corresponding ADC map (**B**) and the whole lesion ADC-histogram (**C**) of GBM in a 74 years old male patient. The evaluated parameters were as follows (ADC parameters all in 10^−5^ mm^2^ × s^−1^): ADCmean: 158.42, ADCmin: 0.01, ADCmax: 303.40, ADCp10: 107.50, ADCp25: 128.80, ADCp75: 184.50, ADCp90: 223.40, ADCmedian 153.30, ADCmodus: 136.40, Kurtosis: 2.96, Skewness: 0.37, Entropy: 5.10. The inferior row shows a representative axial T1 weighted post contrast image (**D**) of brain abscess in a 71 years old female patient, the corresponding axial ADC map (**E**) and the respective whole lesion ADC-histogram (**F**). The evaluated parameters were as follows (ADC parameters all in 10^−5^ mm^2^ × s^−1^): ADCmean: 107.11, ADCmin 5.80, ADCmax: 287.70, ADCp10: 63.70, ADCp25: 73.40, ADCp75: 134.50, ADCp: 174.80, ADCmedian: 94.20, ADCmodus: 73.40, Kurtosis: 2.79, Skewness: 0.85, Entropy: 4.81.

**Figure 2:**
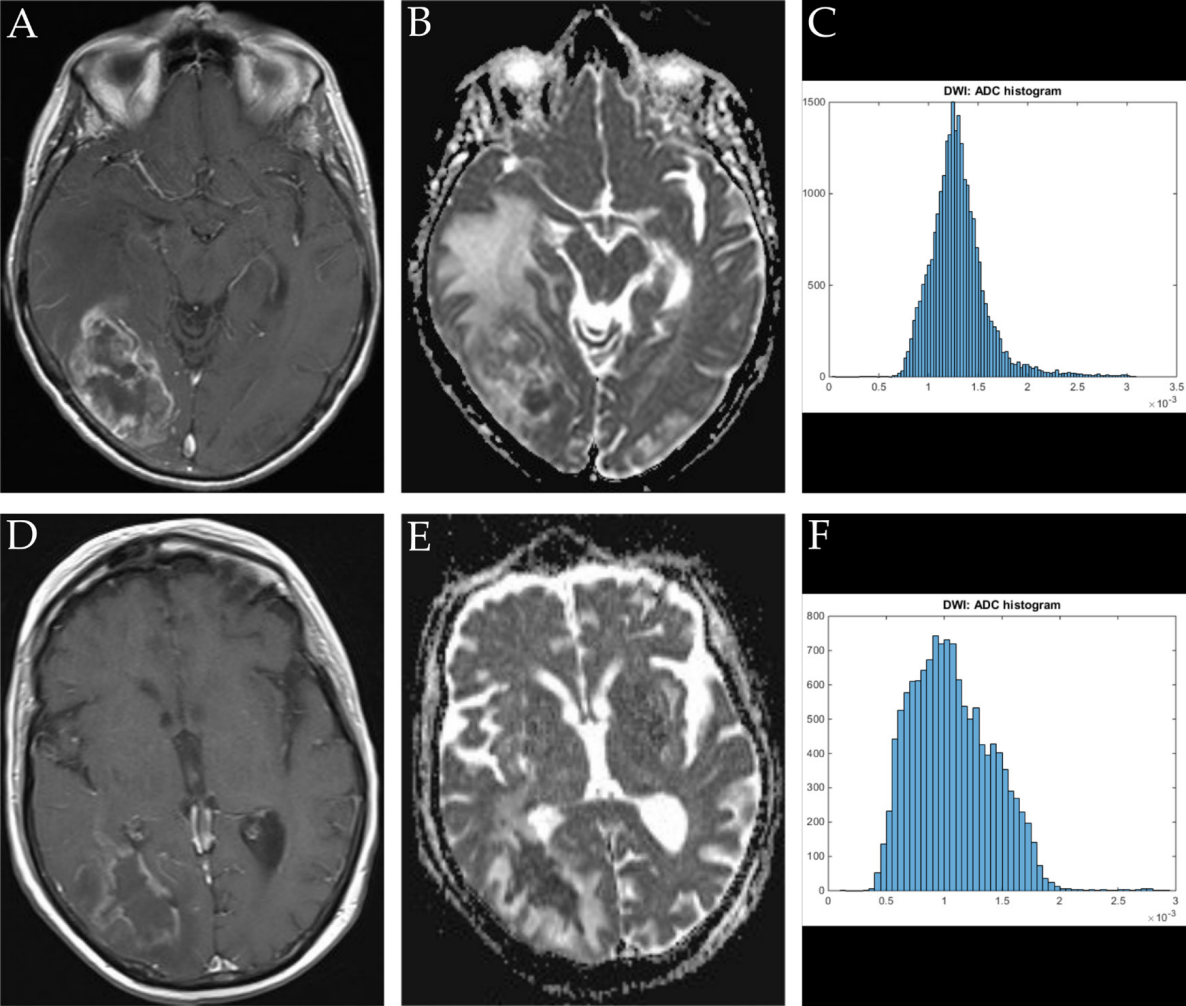
Imaging findings and corresponding ADC-histograms of morphologically resembling temporo-occipital manifestations of GBM and brain abscess The upper row shows a representative axial T1 weighted post contrast image (**A**), the corresponding ADC map (**B**) and the whole lesion ADC-histogram (**C**) of GBM in a 72 years old female patient. Evaluated parameters were as follows (ADC parameters all in 10^−5^ mm^2^ × s^−1^): ADCmean: 125.20, ADCmin: 41.40, ADCmax: 309.30, ADCp10: 82.20, ADCp25: 99.70, ADCp75 148.90, ADCp90: 163.10, ADCmedian: 125.20, ADCmodus: 150.20, Kurtosis: 4.01, Skewness: 0.51, Entropy: 1.32. The inferior row shows a representative axial T1 weighted post contrast image (**D**) of brain abscess in a 74 years old female patient, the corresponding axial ADC map (**E**) and the respective whole lesion ADC-histogram (**F**). Evaluated parameters were as follows (ADC parameters all in 10^−5^ mm^2^ x s^−1^): ADCmean: 107.22, ADCmin: 10.60, ADCmax: 291.90, ADCp10: 63.95, ADCp25: 80.03, ADCp75: 131.00, ADCp90: 155.60 x, ADCmedian: 103.40, ADCmodus 99.10, Kurtosis: 3.36, Skewness: 0.56, Entropy: 4.75.

**Figure 3:**
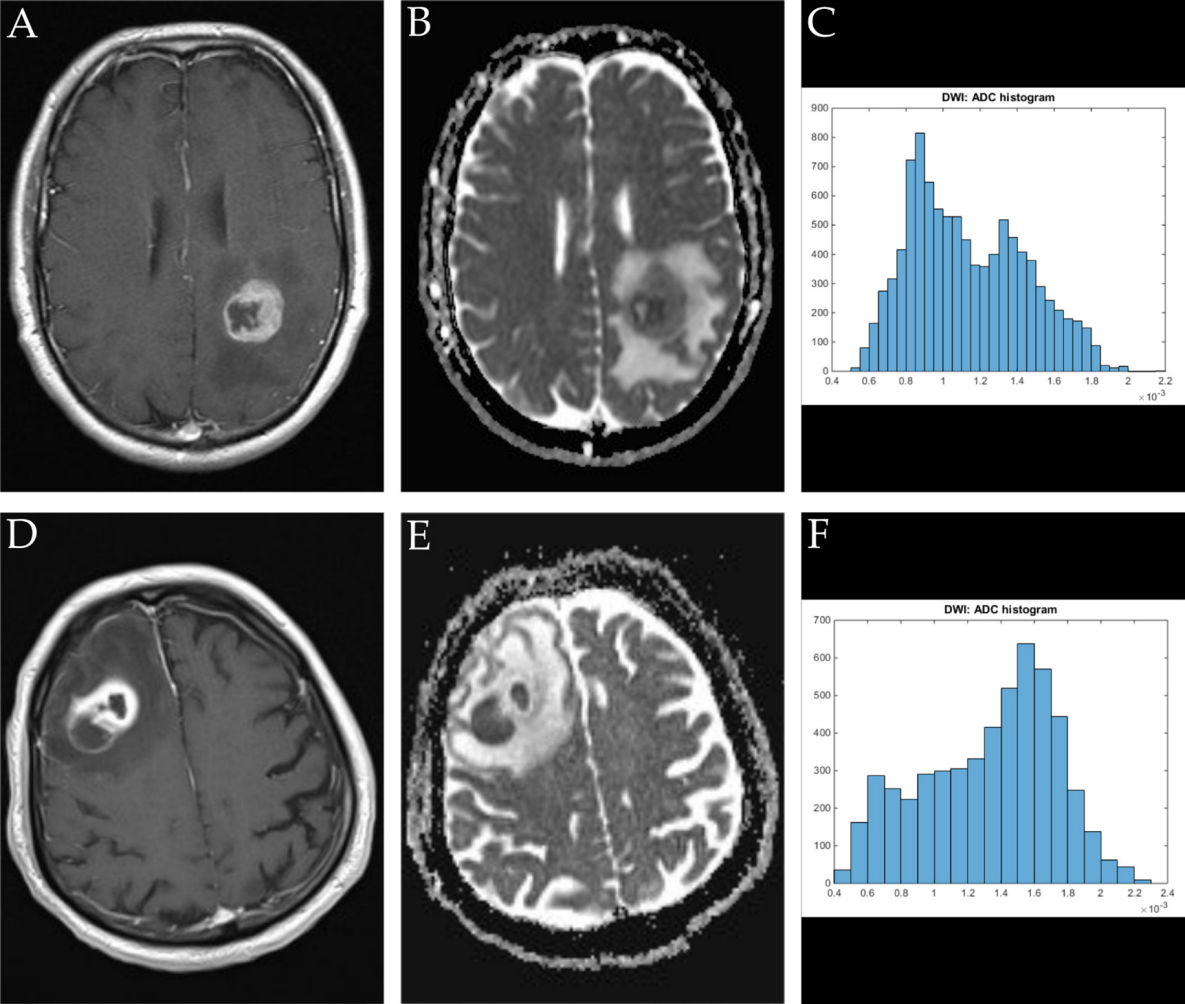
Imaging findings and corresponding ADC-histograms of morphologically resembling manifestations of GBM (left-sided parietal) and brain abscess (right-sided dorso-lateral prefrontal cortex) The upper row shows a representative axial T1 weighted post contrast image (**A**), the corresponding ADC map (**B**) and the whole lesion ADC-histogram (**C**) of GBM in a 53 years old male patient. The evaluated parameters were as follows (ADC parameters all in 10^−5^ mm^2^ × s^−1^): ADCmean: 113.21, ADCmin: 52.30, ADCmax: 217.40, ADCp10: 76.70, ADCp25: 87.70, ADCp75: 136.80, ADCp90: 157.00, ADCmedian: 108.30, ADCmodus: 89.00, Kurtosis: 2.25, Skewness: 0.38, Entropy 4.55. The inferior row shows a representative axial T1 weighted post contrast image (**D**) of brain abscess in a 80 years old male patient, the corresponding axial ADC map (**E**) and the respective whole lesion ADC-histogram (**F**). Evaluated parameters were as follows (ADC parameters all in 10^−5^ mm^2^ × s^−1^): ADCmean: 133.02, ADCmin: 46.60, ADCmax: 224.80, ADCp10: 71.80, ADCp25: 101.95, ADCp75: 163.00, ADCp90: 179.39, ADCmedian: 141.00, ADCmodus: 160.90, Kurtosis: 2.20, Skewness: −0.33, Entropy: 3.92.

ADC volume histogram analysis gave the following values for the overall collective (each median (range) and mean value ± standard deviation, all in x 10^−5^ mm^2^ x s^−1^): ADCmin = 24.700 (101.20), 27.39 ± 25.94, ADCmean = 120.66 (148.53), 124.36 ± 31.18, ADCmax = 283.70 (290.50), 275.06 ± 55.81, ADCp10 = 77.60 (341.40), 80.89 ± 37.18, ADCp25 = 92.65 (110.60), 93.68 ± 25.40, ADCp75 = 147.50 (200.30), 149.95 ± 42.63, ADCp90 = 172.63 (218.18), 199.36 ± 203.90, ADCmedian = 117.40 (875.20), 127.13 ± 86.37, ADCmodus = 106.30 (282.20), 111.41 ± 51.29. Evaluation of histogram-based characteristics of the investigated volumes for the overall collective gave the following values (each median (range) and mean value ± standard deviation): skewness = 0.581 (5.86), 0.69 ± 0.79, entropy = 4.59 (2.59), 4.49 ± 0.59 and kurtosis = 3.10 (22.19), 4.10 ± 3.27. Figures 1, 2 and 3 provide examples of ADC maps (B, E) coregistered to corresponding T1 weighted post contrast images (A, D) and the respective whole lesion histograms (C, F) of morphologically similar appearing GBM and BA in frequently encountered locations. Table 1 summarizes the findings in the overall collective.

**Table 1:** Summarizes ADC values and histogram analysis parameters of all investigated lesions

Parameter	Mean ± Standard deviation	Median	Range
**ADCmin**	0.00027391 ± 0.00025943	0.000247	0.001012
**ADCmean**	0.00124356 ± 0.00031182	0.00120664	0.00148531
**ADCmax**	0.0027506 ± 0.00055808	0.002837	0.002905
**ADCp10**	0.00080898 ± 0.00037178	0.000776	0.003414
**ADCp25**	0.00093688 ± 0.00025404	0.0009265	0.001106
**ADCp75**	0.00149951 ± 0.00042632	0.001475	0.002003
**ADCp90**	0.00199369 ± 0.00203903	0.0017263	0.0210818
**ADCmedian**	0.00127128 ± 0.00086373	0.001174	0.008752
**ADCmodus**	0.0011141 ± 0.0005129	0.001063	0.002822
**Skewness**	0.69296671 ± 0.78509629	0.58084081	5.86608566
**Entropy**	4.4994238 ± 0.58859579	4.59820438	2.59501778
**Kurtosis**	4.09263681 ± 3.2709259	3.0986996	22.1909068
**Lesion volume (ml)**	41.58821 ± 37.82949	29.614	170.887

### Group comparisons

**Figure 4:**
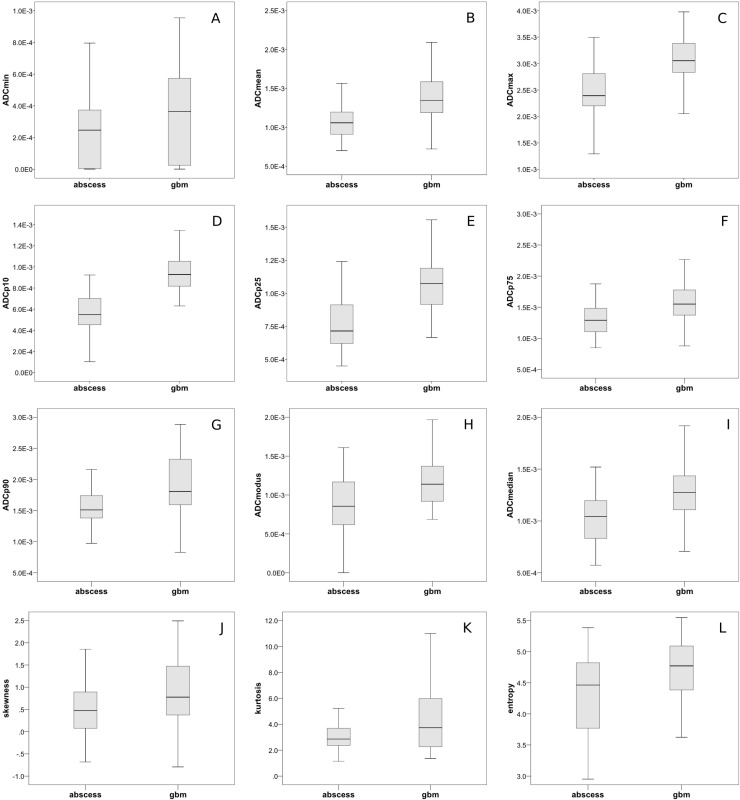
Group comparisons of ADC-histogram parameters and histogram characteristics (**A**–**I**) graphically demonstrates differences in the respective evaluated ADC-histogram parameters using boxplots to compare BA and GBM. All differences displayed reached statistical significance (according *p*-values are given in Table 2). (**J**–**L**) compares ADC-histogram characteristics between GBM and brain abscess. Skewness, kurtosis and entropy were significantly lower in abscesses compared to GBM (according *p*-values are given in Table 2).

Homoscedasticity by means of Levene's Test was identified for the following parameters (corresponding *p*-values are given in brackets): lesion volume (*p* < 0.001), ADCmin (*p* < 0.001), ADCmean (*p* = 0.013), ADCp75 (*p* = 0.001), ADCp90 (*p* = 0.004) and kurtosis (*p* < 0.001). Hence, unpaired *t*-test was performed to compare values between the brain abscess and the GBM group for this set of parameters. Heteroscedasticity was identified for the following parameters (corresponding *p*-values of Levene`s Test for homogeneity of variances are given in brackets): ADCmax (*p* = 0.770), ADCp10 (*p* = 0.754), ADCp25 (*p* = 0.801), ADCmodus (*p* = 0.421), ADCmedian (*p* = 0.088), skewness (*p* = 0.120) and entropy (*p* = 0.072). Hence, Mann-Whitney-*U* Test was performed to compare values between the aforementioned groups for the latter set off parameters. Table 2 compares all investigated parameters in brain abscess patients and GBM patients. Corresponding *p*-Values of the according significance tests are also displayed. In brief, statistically significant differences were identified for lesion volume, all ADC fractions and histogram-based parameters as well. The overall lesion volume was significantly smaller in BA compared to GBM. ADC histogram parameter values in general were significantly lower in BA compared to GBM. Furthermore, analysis of histogram-based VOI characteristics demonstrated significantly decreased skewness, kurtosis and entropy in BA in comparison to GBM. Figure 4A–4L gives the boxplots graphically summarizing differences in evaluated parameters when comparing BA and GBM.

**Table 2:** Compares estimated values of GBMs and abscesses and gives the corresponding results of the statistical group comparison tests

Parameter	Mean ± Standard Deviation		Median (Minimum–Ma×imum)		*p*-value
	GBM	Abscess	GBM	Abscess	
**ADCmin** **× 10^−5^ mm^2^ × s^−1^**	32.69 ± 28.24	22.44 ± 20.16	36.40 (0.1-95.5)	24.7 (0.01-79.6)	**0.037**
**ADCmean** **× 10^−5^ mm^2^ × s^−1^**	138.79 ± 32.22	105.02 ± 20.36	134.43 (72.01–218.48)	105.69 (69.95–156.36)	**<0.001**
**ADCma×** **× 10^−5^ mm^2^ × s^−1^**	298.57 ± 53.04	247.17 ± 48.73	305.45 (107.30–397.80)	239.2 (129.2–349.60)	**<0.001**
**ADCp10** **× 10^−5^ mm^2^ × s^−1^**	93.53 ±16.69	57.16 ± 16.67	92.85 (63.10–141.10)	54.88 (10.30–92.63)	**<0.001**
**ADCp25** **× 10^−5^ mm^2^ × s^−1^**	107.96 ± 21.00	75.73 ± 19.82	107.45 (66.60–155.60)	71.50 (45.00–124.00)	**<0.001**
**ADCp75** **× 10^−5^ mm^2^ × s^−1^**	165.37 ± 49.64	129.10 ± 24.80	155.05 (75.30–275.60)	129.00 (84.90–187.50)	**<0.001**
**ADCp90** **× 10^−5^ mm^2^ × s^−1^**	193.28 ± 53.02	159.37 ± 35.91	180.63 (82.79–288.40)	151.10 (97.40–248.00)	**<0.001**
**ADCmedian** **× 10^−5^ mm^2^ × s^−1^**	132.22 ± 34.68	117.29 ± 118.63	127.55 (70.60–25.58)	104.30 (57.30–93.25)	**<0.001**
**ADCmodus** **× 10^−5^ mm^2^ × s^−1^**	130.48 ± 58.12	88.78 ± 34.91	113.90 (68.70–28.23)	85.50 (0.01-160.90)	**<0.001**
**Skewness**	0.88 ± 0.89	0.53 ± 0.61	0.78 (−2.07–0.49)	0.47 (−0.68–2.14)	**0.010**
**Entropy**	4.69 ± 0.52	4.31 ±0.58887276	4.77 (3.2–4.30)	4.46 (2.95–5.38)	**0.002**
**Kurtosis**	5.05 ± 4.21	3.22 ± 1.36	3.74 (1.35–3.11)	2.86 (1.15–8.55)	**0.004**
**Lesion volume (ml)**	59.82 ± 40.57	23.71 ± 24.29	51.33 (37.15 −171.86)	17.674 (0.97-121.25)	**<0.001**

Results achieving statistical significance are displayed in bold writing.

### ROC curve analysis

**Figure 5:**
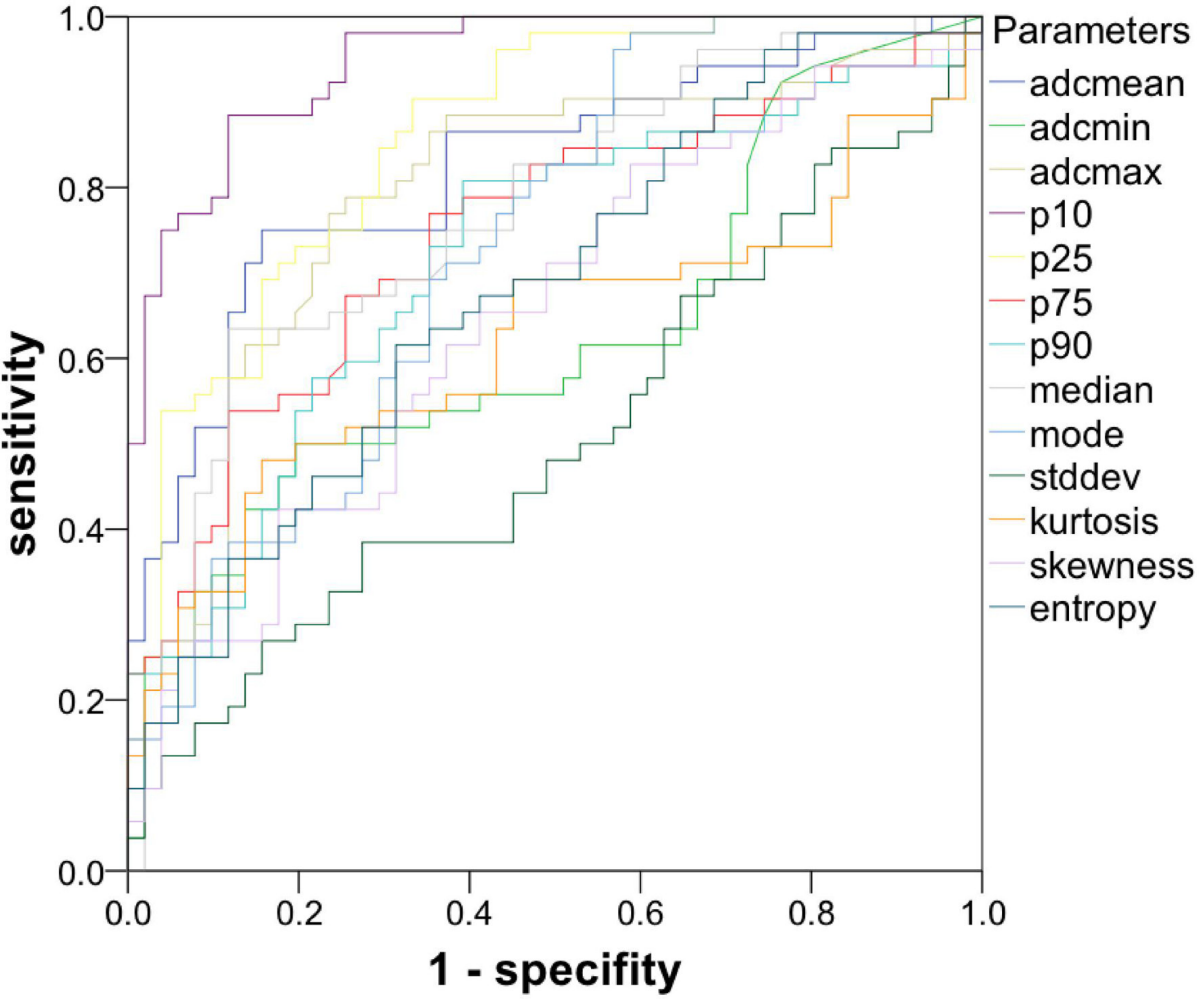
ROC curves of the investigated parameters gives the ROC curves of ADC-histogram parameters and ADC-histogram characteristics ADCp10 revealed the highest level of accuracy regarding the discriminability between GBM and abscess.

Figure 5 summarizes the ROC curves. AUC values were calculated for each of the evaluated parameters and are given in categories according their respective level of accuracy in the following paragraph. Parameters only achieving a poor level of accuracy (AUC: 0.5–0.6) were ADC-histogram kurtosis: AUC = 0.622, ADCmin: AUC = 0.627, ADC-histogram skewness: AUC = 0.648 and ADC histogram entropy: AUC = 0.680. Parameters achieving a fair level of accuracy (AUC 0.7-0.8) were ADCp90: AUC = 0.717, ADCmodus: AUC = 0.731, ADCp75 AUC = 0.747, ADCmedian: AUC = 0.772, ADCmax: AUC = 0.791. Parameters achieving a good level of accuracy (AUC 0.7-0.8) were ADCmean: AUC = 0.821 and ADCp25: AUC = 0.862. The only parameter showing an excellent level of accuracy was ADCp10: AUC = 0.948.

### Cut-point estimation

In case of AUC values greater than 0.8, Youden's index was used to estimate optimal cut-points for the respective parameters [30]. The following values were identified; ADCp10: 70 × 10^−5^ mm^2^ × s^−1^ (sensitivity: 0.98, specificity: 0.75), ADCmean: 120 × 10^−5^mm^2^ × s^−1^ (sensitivity 0.75, specificity 0.84) and ADCp25: 90 × 10^−5^mm^2^ × s^−1^ (sensitivity: 0.85, specificity: 0.71). Values lying above the estimated cut-points indicate that the lesion in question is a GBM, vice versa, values lying below the estimated cut-points indicate that the lesion in question is a brain abscess.

### Correlative analysis

Calculation of Spearman's Rank Order correlation for lesion size and histogram profiling parameters gave the following results: – lesion volume correlated significantly with ADCmean: *r* = 0.337, *p* < 0.001; ADCmax: *r* = 0.474, *p* < 0.001; ADCp10: 0.284, *p* = 0.004; ADCp25: *r* = 0.257, *p* = 0.009; ADCp75: *r* = 0.354, *p* < 0.001; ADCp90: *r* = 305, *p* = 0.002; ADCmodus *r* = 0.327, *p* = 0.001 and Entropy: *r* = 0.503, *p* < 0.001. The strongest correlation is demonstrated in Supplementary Figure 1 via dot plot.

## DISCUSSION

This study aimed to investigate, whether whole lesion ADC histogram profiling has the potential to differentiate between GBM and BA, both presenting as REL with similar appearance patterns in conventional MRI. To our best knowledge, this is the first work showing significant differences in whole lesion ADC histogram parameters and histogram-based characteristics when comparing both entities.

In detail, all parameters investigated revealed statistically significant differences between GBM and BA. However, among the tested variables, ADCp10 was the solitary parameter revealing an excellent level of accuracy regarding the differentiation of both entities, represented by an area under the ROC curve of 0.948. Using a cutpoint value of 70 × 10^−5^mm^2^ × s^−1^, ADCp10 can differentiate between GBM (higher values) and BA (lower values) with a sensitivity of 0.98 and specificity of 0.75.

The potential of ADCp10 as an imaging biomarker has been investigated in few other studies so far. Choi *et al*. exemplarily demonstrated that ADCp10 values differ significantly between atypical GBM and primary central nervous system lymphoma [5]. Furthermore, it was shown that cervical cancer limited to a local disease stage had significantly higher ADCp10 values compared to cervical cancer, which already had gained the ability to metastasize via the lymphatic system and therefore might be used to predict macroscopically undeterminable metastatic lymph node disease [28]. In the context of these previous studies and our current work, we hypothesize that ADCp10 is superior to conventional MRI and the standardly applied ADC measurements regarding the reflection of micro-architectural properties of tissues, and hence, the discrimination of morphologically resembling diseases like GBM and BA. Synthesizing the current knowledge on ADCp10, the parameter probably has the potential to depict a characteristic facet of the diffusion profile of pathologic lesions and consequentially should be investigated more closely in the context of other malignant entities concerning its value as DWI biomarker.

Secondary to ADCp10, ADCp25 and ADCmean were the only two parameters showing a good level of accuracy (0.862 and 0.821, respectively) regarding the discriminability of GBM and BA. The value of ADCp25 as imaging biomarker is not well established yet, however, recently it was demonstrated that ADCp25, in addition to ADCp10, was significantly lower in dedifferentiated types of cervical cancer, which already had developed distant metastases [28]. From a current perspective, it is certainly possible that the lower percentils, ADCp10 and ADCp25, provide concurrent information. It is yet to be determined, whether ADCp25 provides additional data beyond ADCp10 as an imaging biomarker, therefore, further studies investigating ADCp25 and comparing it to ADCp10 are warranted. Contrarily, the role of ADCmean as imaging biomarker is firm. The correlation between ADCmean and the corresponding cellularity of tumors is well investigated [31], and the coherence of ADCmean to the proliferative activity of malignant and benign tumors is increasingly acknowledged [19, 20, 32]. Since simple ADCmean measurements can be performed using most PACS systems, the quantification of whole lesion ADCmean values should be performed including all slices representing the unaccounted lesion in question in case histogram analysis is not available on-site, thus providing a valuable approximation of its complex diffusion profile.

Interestingly, histogram-based characteristics (skewness, kurtosis, entropy) describing the distribution of ADC values and the resulting shape of the histogram only achieved poor values of accuracy regarding the differentiation between both investigated entities. Skewness and kurtosis are believed to reflect the cellular architecture of different malignant tumors to varying extent and have successfully been used to discriminate between microscopically and prognostically different subgroups of malignant tumors [27, 29]. Both parameters have also been used to assess response of malignant tumors to anti-proliferative treatment regimens [33, 34]. Entropy is believed to primarily represent tumor heterogeneity of malignant lesions [27]. In cervical cancer, loss of p53, which is associated with incremental dedifferentiation and molecular alteration towards a more malignant phenotype, correlated inversely with ADCentropy [28]. Although all ADC histogram-based characteristics were significantly different comparing GBM and BA, the differences were not sufficiently distinct to reliably differentiate between both entities. We therefore conclude that the differences within the respective diffusion profiles of both entities are better reflected by ADCp10. Our study suffers from some limitations. Firstly, it is of retrospective nature. Secondly, it only includes patients investigated on a single MRI scanner of a single center. As a consequence, further, ideally prospective studies including different scanner types (1.5Tesla, 3Tesla, different vendors) and DWI sequences (e.g. RESOLVE, TSE, standard EPI) are necessary to validate our findings in a greater cohort comparing different scanner types. Furthermore, only the two most common entities presenting as REL were included. Future investigations should also include a considerable number of patients with brain metastases and primary central nervous system lymphoma to investigate the value of ADC histogram profiling for differentiation of further entities presenting as REL.

## MATERIALS AND METHODS

### Ethics approval

The study was approved by the ethics committee of the medical council of Baden-Württemberg (Ethik-Kommission Landesärtzekammer Baden-Württemmberg, F-2017-047).

### Patients selection

Potential patients in the period of 2012 through 2017 were identified on the basis of the diagnoses ‚GBM’ and ‚brain abscess’ conducting a full-text search in the database of the institute for neuroradiology. The search revealed a total of 484 patients in our radiological database, who had both differentials as probable diagnosis in the report of the respective cMRI study. The corresponding histopathological examinations revealed that 395 patients had GBM, 89 patients had pyogenic BA. All cases were reviewed by two experienced radiologists (DHR., AS) with more than 10 years of experience in the neuro-oncological setting. In 381 cases, consensus was found between both radiologists and the radiological diagnosis matched the histopathological diagnosis. In 103 cases, imaging features that commonly discriminate between both entities [35] were inconclusive for both readers and therefore included in our study.

Only previously untreated patients with pretreatment MRI inclusive of DWI were included. None of the cases included showed significant hemorrhage or calcifications. Scans showing significant motion artifacts were excluded. Neuropathological confirmation of the diagnosis was present in all cases. In total 103 patients were included (3 to 89 years). 51 patients (18 female, 34 male, mean age: 59 years) 51 patients (18 female, 34 male, mean age: 59 years) with BA and 52 patients (22 female, 31 male, mean age: 65) with GBM were included in the study. None of the studied patients were immune-deficient. Supplementary Table 1 summarizes demographics and clinical characteristics of the investigated patients.

### MR imaging

For all patients MRI of the brain was performed using a 1.5 T device (MAGNETOM Aera, Tx/Rx CP head coil, Siemens, Erlangen, Germany). The imaging protocol included the following sequences:

1. axial T2 weighted (T2w) turbo spin echo (TSE) sequence (TR/TE: 4000/69, flip angle: 150°, slice thickness: 4mm, acquisition matrix: 200×222, field of view: 100mm);

2. axial T1 weighted (T1w) turbo spin echo (TSE) sequences (TR/TE: 765/9.5, flip angle: 150°, slice thickness: 5mm, acquisition matrix: 200×222, field of view: 100mm) prior and post intravenous application of contrast medium (gadopentate dimeglumine, Magnevist®, Bayer Schering Pharma, Leverkusen, Germany)

3. axial DWI (EPI sequence; TR/TE: 5400/69, flip angle 180°, slice thickness: 4mm, acquisition matrix: 200×222, field of view: 100mm) with b values of 0, 500 and 1000 s/mm^2^. ADC maps were generated automatically by the implemented software package.

All images were available in digital form and analyzed by two experienced radiologists (DHR, SS) without knowledge of the histopathological diagnosis on a PACS workstation (Impax EE R20 XII).

Figures 1, 2, 3A show representative axial T1 weighted post contrast images of GBM. Figures 1, 2, 3D show representative axial T1 weighted post contrast images of brain abscess. Figures 1, 2, 3B and 3E show corresponding representative axial ADC images of the respective lesions.

### Histogram profiling of ADC values

DWI data was transferred in DICOM format and processed offline with a custom-made Matlab-based application (The Mathworks, Natick, MA) on a standard windows operated system. The ADC maps and T1 post contrast images were displayed within a graphical user interface (GUI), which enables the reader to scroll through the slices and draw a volume of interest (VOI) at the tumor's boundary. The VOI was created by manually drawing regions of interest (ROIs) along the margin of the contrast enhancing portion of the lesion on the T1 weighted images, using all slices displaying the lesion (whole lesion measure). Coregistration to the corresponding ADC maps was performed automatically by the application. Successful coregistration was verified by the readers in all cases. All measures were performed by two authors (DHR, AS). Inter-reader-reliability was excellent (*r* = 0.964 using Pearson correlation) and the results of the histogram analysis of both readers were averaged for further analysis. The ROIs were modified in the GUI and saved (in Matlab-specific format) for later processing. After setting the ROIs, the following parameters were calculated and given in a spreadsheet format: Total ROI volume (cm^3^), mean (ADCmean), maximum (ADCmax), minimum (ADCmin), median (ADCmedian), modus (ADCmodus) and the percentils: 10th (ADCp10), 25th (ADCp25), 75th (ADCp75) and 90th (ADCp90). Additionally, ADC-histogram based characteristics of the VOI - kurtosis, skewness and entropy – were computed. All calculations were performed using in-build Matlab functions. All ADC images showing the respective lesion were used to issue the whole lesion ADC-histogram. Examples of ADC-histograms corresponding to the lesions shown in Figures 1, 2, 3A, 3B, 3D and 3E are given in Figures 1, 2, 3C and 3F.

### Statistical analysis

Statistical analysis was performed using IBM SPSS 23™ (SPSS Inc., Chicago, IL, USA). In a first step, collected data was evaluated by means of descriptive statistics. In a second step, Levene's Test for homogeneity of variance (homoscedasticity) was performed to assess the equality of variances of ADC values and ADC derived histogram parameters in the groups of GBM patients and brain abscess patients. Subsequently, the suitable test for group comparisons was identified. In case of homoscedasticity, unpaired *t* test was performed to compare values among both groups. In case of inhomogeneity of variance (heteroscedasticity), Mann-Whitney-*U* test was performed to compare values among the different groups. Group comparisons were performed for all obtained ADC parameters and histogram values, comparing GBMs and BAs.

Finally, to assess the accuracy of ADC volume histogram profiling, receiver operating characteristics (ROC) curve analysis was performed and the respective area under the curve (AUC) was calculated. For identification of optimal cut-point values discriminating between GBM and BA, Youden's Index was applied for parameters with fair (AUC: 70–80), good (AUC: 80–90) and excellent (AUC: 90-1) accuracy.

Additionally, correlative analysis using Spearman's Rank Order correlation was performed to investigate associations between lesion size and diffusion histogram profiling parameters.

## CONCLUSIONS

Conventional MRI is a very suitable tool to morphologically characterize a space occupying intracranial lesion. Unfortunately, in a number of cases, anatomical imaging alone is insufficient to differentiate GBM and BA, for the fact that both diseases appear as resembling REL with similar perifocal edema and a central cystic compartment. DWI performing ADC histogram profiling provides unique clues to the structural features and geometric organization of mass lesions and has therefore proven superior to conventional MRI and even the standardly applied ADC quantification. All investigated parameters exhibited statisically significant differences in comparison of GBM and BA. However, only ADCp10 could reliably distinguish GBM from BA in our study. In conclusion we recommend to include ADC histogram profiling into the differential diagnostic process for the differentiation of REL in the clinical setting.

## SUPPLEMENTARY MATERIALS FIGURES AND TABLES





## Abbreviations

GBM: glioblastoma multiforme
BA: brain abscess
ADC: apparent diffusion coefficient
DWI: diffusion weighted imaging
REL: ring enhancing lesion
MRI: magnetic resonance imaging
BBB: blood-brain-barrier

## References

[1] Dibble EH, Boxerman JL, Baird GL, Donahue JE, Rogg JM (2017). Toxoplasmosis versus lymphoma: Cerebral lesion characterization using DSC-MRI revisited. Clinical Neurology and Neurosurgery.

[2] Skovrlj B, Rasouli J, Caridi J, Taylor WM, Galyon DD (2014). Progressive multifocal leukoencephalopathy presenting as a single ring-enhancing lesion. Clinical Neurology and Neurosurgery.

[3] Britton PN, Chaseling R (2012). Brain abscess in a recent immigrant. J Paediatr Child Health.

[4] Santosh V, Mahadevan A, Chickabasaviah YT, Bharath RD, Krishna SS (2010). Infectious lesions mimicking central nervous system neoplasms. Seminars in Diagnostic Pathology.

[5] Choi YS, Lee HJ, Ahn SS, Chang JH, Kang SG, Kim EH, Kim SH, Lee SK (2017). Primary central nervous system lymphoma and atypical glioblastoma: differentiation using the initial area under the curve derived from dynamic contrast-enhanced MR and the apparent diffusion coefficient. Eur Radiol.

[6] Kumar Garg R, Kumar Singh M, Misra S (2000). Single-enhancing CT lesions in Indian patients with seizures: a review. Epilepsy Research.

[7] Verma R, Gupta R (2014). Multiple ring-enhancing lesions: diagnostic dilemma between neurocysticercosis and tuberculoma. BMJ Case Rep.

[8] Fuchsmann C, Traverse-Glehen A, Durbec M, Dubreuil C, Tringali S (2010). Glioblastoma multiforme mimicking a frontal abscess after surgery for a large vestibular schwannoma. European Annals of Otorhinolaryngology, Head and Neck Diseases.

[9] Cianfoni A, Calandrelli R, De Bonis P, Pompucci A, Lauriola L, Colosimo C (2010). Nocardia brain abscess mimicking high-grade necrotic tumor on perfusion MRI. J Clin Neurosci.

[10] Omuro AM, Leite CC, Mokhtari K, Delattre JY (2006). Pitfalls in the diagnosis of brain tumours. Lancet Neurol.

[11] Law M, Hamburger M, Johnson G, Inglese M, Londono A, Golfinos J, Zagzag D, Knopp EA (2004). Differentiating surgical from non-surgical lesions using perfusion MR imaging and proton MR spectroscopic imaging. Technol Cancer Res Treat.

[12] Li YM, Suki D, Hess K, Sawaya R (2016). The influence of maximum safe resection of glioblastoma on survival in 1229 patients: Can we do better than gross-total resection?. Journal of Neurosurgery.

[13] Patel K, Clifford DB (2014). Bacterial brain abscess. Neurohospitalist.

[14] Faehndrich J, Weidauer S, Pilatus U, Oszvald A, Zanella FE, Hattingen E (2011). Neuroradiological viewpoint on the diagnostics of space-occupying brain lesions. Clin Neuroradiol.

[15] Upadhyay N, Waldman AD (2011). Conventional MRI evaluation of gliomas. BJR.

[16] Toh CH, Wei KC, Ng SH, Wan YL, Lin CP, Castillo M (2011). Differentiation of Brain Abscesses from Necrotic Glioblastomas and Cystic Metastatic Brain Tumors with Diffusion Tensor Imaging. AJNR Am J Neuroradiol.

[17] Toh CH, Wei KC, Chang CN, Ng SH, Wong HF, Lin CP, Sherman J (2014). Differentiation of brain abscesses from glioblastomas and metastatic brain tumors: comparisons of diagnostic performance of dynamic susceptibility contrast-enhanced perfusion MR imaging before and after mathematic contrast leakage correction. PLoS ONE.

[18] Le Bihan D (2013). Apparent diffusion coefficient and beyond: what diffusion MR imaging can tell us about tissue structure. Radiology.

[19] Schob S, Voigt P, Bure L, Meyer HJ, Wickenhauser C, Behrmann C, Höhn A, Kachel P, Dralle H, Hoffmann KT, Surov A (2016). Diffusion-Weighted Imaging Using a Readout-Segmented, Multishot EPI Sequence at 3 T Distinguishes between Morphologically Differentiated and Undifferentiated Subtypes of Thyroid Carcinoma-A Preliminary Study. TRANON.

[20] Schob S, Meyer J, Gawlitza M, Frydrychowicz C, Müller W, Preuss M, Bure L, Quäschling U, Hoffmann KT, Surov A (2016). Diffusion-Weighted MRI Reflects Proliferative Activity in Primary CNS Lymphoma. PLoS ONE.

[21] Schob S, Surov A, Wienke A, Meyer HJ, Spielmann RP, Fiedler E (2017). Correlation Between Aquaporin 4 Expression and Different DWI Parameters in Grade I Meningioma. Mol Imaging Biol.

[22] Tomar V, Yadav A, Rathore RKS, Verma S, Awasthi R, Bharadwaj V, Ojha BK, Prasad KN, Gupta RK (2011). Apparent diffusion coefficient with higher b-value correlates better with viable cell count quantified from the cavity of brain abscess. American Journal of Neuroradiology.

[23] Reddy JS, Mishra AM, Behari S, Husain M, Gupta V, Rastogi M, Gupta RK (2006). The role of diffusion-weighted imaging in the differential diagnosis of intracranial cystic mass lesions: a report of 147 lesions. Surgical Neurology.

[24] Lee EJ, Ahn KJ, Ha YS, Oh HE, Park CS, Song SY, Park NH, Kim MS (2006). Unusual findings in cerebral abscess: report of two cases. BJR.

[25] Hakyemez B, Erdogan C, Yildirim N, Parlak M (2005). Glioblastoma multiforme with atypical diffusion-weighted MR findings. BJR. British Institute of Radiology.

[26] Reiche W, Schuchardt V, Hagen T, Il'yasov KA, Billmann P, Weber J (2010). Differential diagnosis of intracranial ring enhancing cystic mass lesions--role of diffusion-weighted imaging (DWI) and diffusion-tensor imaging (DTI). Clinical Neurology and Neurosurgery.

[27] Just N (2014). Improving tumour heterogeneity MRI assessment with histograms. British Journal of Cancer.

[28] Schob S, Meyer HJ, Pazaitis N, Schramm D, Bremicker K, Exner M, Höhn AK, Garnov N, Surov A (2017). ADC Histogram Analysis of Cervical Cancer Aids Detecting Lymphatic Metastases—a Preliminary Study. Mol Imaging Biol.

[29] Schob S, Meyer H, Dieckow J, Pervinder B, Pazaitis N, Höhn A, Garnov N, Horvath-Rizea D, Hoffmann KT, Surov A (2017). Histogram Analysis of Diffusion Weighted Imaging at 3T is Useful for Prediction of Lymphatic Metastatic Spread, Proliferative Activity, and Cellularity in Thyroid Cancer. IJMS. Multidisciplinary Digital Publishing Institute.

[30] Ruopp M, Perkins N, Whitcomb B, Schisterman E (2008). Youden Index and Optimal Cut-Point Estimated from Observations Affected by a Lower Limit of Detection. WILEY-VCH Verlag.

[31] Chen L, Liu M, Bao J, Xia Y, Zhang J, Zhang L, Huang X, Wang J, Hess CP (2013). The Correlation between Apparent Diffusion Coefficient and Tumor Cellularity in Patients: A Meta-Analysis. PLoS ONE.

[32] Surov A, Caysa H, Wienke A, Spielmann RP, Fiedler E (2015). Correlation Between Different ADC Fractions, Cell Count, Ki-67, Total Nucleic Areas and Average Nucleic Areas in Meningothelial Meningiomas. Anticancer Res.

[33] King AD, Chow KK, Yu KH, Mo FKF, Yeung DKW, Yuan J, Bhatia KS, Vlantis AC, Ahuja AT (2013). Head and neck squamous cell carcinoma: diagnostic performance of diffusion-weighted MR imaging for the prediction of treatment response. Radiology.

[34] Song YS, Choi SH, Park CK, Yi KS, Lee WJ, Yun TJ, Kim TM, Lee SH, Kim JH, Sohn CH, Park SH, Kim IH, Jahng GH (2013). True Progression versus Pseudoprogression in the Treatment of Glioblastomas: A Comparison Study of Normalized Cerebral Blood Volume and Apparent Diffusion Coefficient by Histogram Analysis. Korean J Radiol.

[35] Garg RK, Sinha MK (2010). Multiple ring-enhancing lesions of the brain. Journal of Postgraduate Medicine.

