# Supplementary material for: The value of whole lesion ADC histogram profiling to differentiate between morphologically indistinguishable ring enhancing lesions–comparison of glioblastomas and brain abscesses
Source: Oncotarget. 2018 Apr 6;9(26):18148–59. doi: 10.18632/oncotarget.24454 (PMC5915063; doi:10.18632/oncotarget.24454)
Supplement: Supplementary file 1 [file oncotarget-09-18148-s001.pdf]

The value of whole lesion ADC histogram profiling to differentiate between morphologically indistinguishable ring enhancing lesions–comparison of glioblastomas and brain abscesses

SUPPLEMENTARY MATERIALS

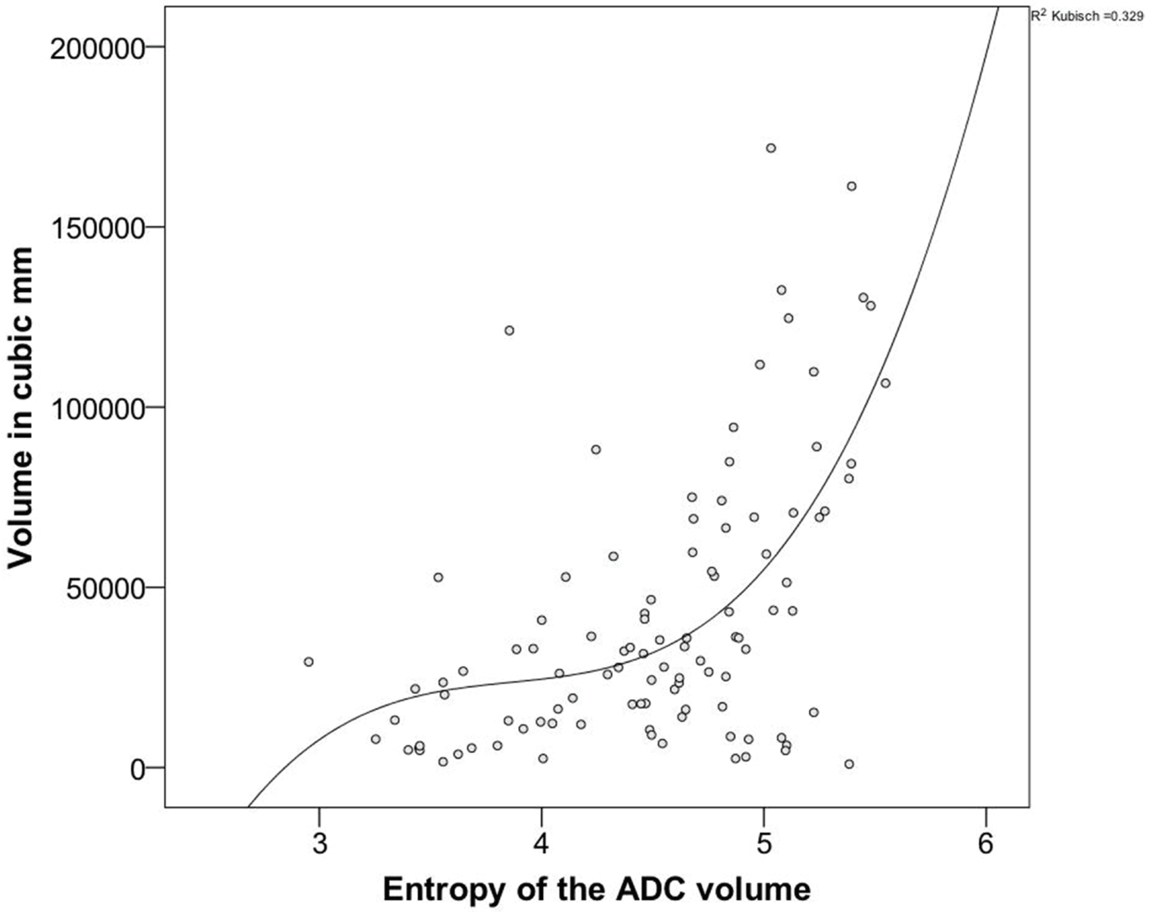

Supplementary Figure 1: Provides the association between lesion size and entropy of the ADC volume. Interestingly, entropy increases significantly with the size of the lesion in question ($r = 0.503$, $p < 0.001$).

Supplementary Table 1: Summarizes clinical data and paraclinical features of all investigated lesions. See Supplementary_Table_1
