# Supplementary material for: The value of whole lesion ADC histogram profiling to differentiate between morphologically indistinguishable ring enhancing lesions–comparison of glioblastomas and brain abscesses
Source: Oncotarget. 2018 Apr 6;9(26):18148–59. doi: 10.18632/oncotarget.24454 (PMC5915063; doi:10.18632/oncotarget.24454)
Supplement: Supplementary file 2 [file oncotarget-09-18148-s002.docx]

**Supplementary Table 1: Ssummarizes clinical data and paraclinical features of all investigated lesions**

| Case | Age | Sex | Result of bacteriological and histological examination | Localisation | Lesion volume  in mL |
| --- | --- | --- | --- | --- | --- |
| 1 | 18 | male | Staphylococcus aureus | Left parietal lobe and thalamus | 82.51 |
| 2 | 50 | male | Streptococcus intermedius | Left parietal lobe | 29.61 |
| 3 | 78 | female | Nocardia | Right occipital lobe | 32.99 |
| 4 | 69 | male | Inconclusive | Right temporal lobe | 25.83 |
| 5 | 56 | male | Inconclusive | Brainstem | 7.83 |
| 6 | 89 | male | Streptococcus intermedius | Right thalamus | 21.68 |
| 7 | 47 | male | Streptococcus constellatus | Left temporal lobe | 5.38 |
| 8 | 51 | male | Candida albicans | Right basal ganglia | 13.16 |
| 9 | 71 | male | Inconclusive | Left temporal lobe | 29.34 |
| 10 | 59 | male | Streptococcus intermedius | Left frontal lobe | 10.74 |
| 11 | 69 | male | Inconclusive | Right frontal lobe | 2.50 |
| 12 | 63 | male | Klebsiella pneumoniae | Left frontal lobe | 6.18 |
| 13 | 32 | male | Inconclusive | Right frontal lobe | 20.23 |
| 14 | 60 | male | Inconclusive | Left parietal lobe | 0.97 |
| 15 | 78 | male | Grampos. rods | Right frontal lobe | 17.80 |
| 16 | 72 | female | Staphylococcus aureus | Left temporal lobe | 6.68 |
| 17 | 57 | female | Salmonella typhimurium | Right frontal lobe | 26.75 |
| 18 | 71 | female | Fusobacterium nucleatum | Right frontal lobe | 11.97 |
| 19 | 73 | female | Propionibacterium avidum | Left occipital lobe | 121.25 |
| 20 | 75 | female | Toxoplasma spec. | Right frontal lobe | 23.45 |
| 21 | 63 | female | Enterococcus faecalis | Left frontal lobe | 21.83 |
| 22 | 76 | male | Staphylococcus haemolyticus | Left frontal lobe | 4.72 |
| 23 | 75 | male | Strepeptococcus intermedius | Right frontal lobe | 12.25 |
| 24 | 3 | male | Streptococcus intermedius | Right frontal lobe | 16.89 |
| 25 | 51 | male | Streptococcus intermedius | Left cerebellum | 26.13 |
| 26 | 57 | female | Fusobacterium nucleatum | Right frontal lobe | 2.50 |
| 27 | 51 | female | Sterptococcus intermedius | Left frontal lobe | 52.76 |
| 28 | 72 | male | Streptococcus anginosus | Right cerebellum | 8.61 |
| 29 | 71 | female | Staphylococcus epidermidis | Left frontal lobe | 58.59 |
| 30 | 73 | female | Streptococcus intermedius | Left temporal lobe | 26.53 |
| 31 | 42 | male | Inconclusive | Left occipital lobe | 15.32 |
| 32 | 81 | male | Inconclusive | Right temporal lobe | 42.75 |
| 33 | 55 | male | Nocardia | Multiple, subcortical lesions in supra- und infratentorial location | 2.96 |
| 34 | 41 | male | Aggregibacter aphrophilus | Left thalamus | 4.76 |
| 35 | 76 | male | Fusobacterium nucleatum | Right frontal lobe | 23.62 |
| 36 | 42 | male | Inconclusive | Right frontal lobe | 25.24 |
| 37 | 75 | female | Inconclusive | Right occipital lobe | 24.29 |
| 38 | 52 | female | Inconclusive | Right temporal lobe | 109.80 |
| 39 | 45 | male | Staphylococcus epidermidis | Right frontal lobe | 41.19 |
| 40 | 65 | male | Fusobacterium nucleatum | Right parietal lobe | 32.85 |
| 41 | 80 | male | Inconclusive | Right occipital lobe | 10.47 |
| 42 | 15 | male | Inconclusive | Right parietal lobe | 27.79 |
| 43 | 64 | male | Pseudomonas aeruginosa | Right temporal lobe | 14.04 |
| 44 | 61 | male | Inconclusive | Right temporal lobe | 4.89 |
| 45 | 50 | male | Listeria monocytogenes | Left thalamus | 5.42 |
| 46 | 36 | female | Inconclusive | Right frontal lobe | 6.02 |
| 47 | 64 | male | Inconclusive | Right frontal lobe | 1.61 |
| 48 | 58 | female | Streptococcus intermedius | Right parietal lobe | 66.47 |
| 49 | 48 | male | Staphylococcus aureus | Left frontal lobe | 17.55 |
| 50 | 75 | male | Proteus mirabilis | Right frontal lobe | 33.55 |
| 51 | 67 | male | Staphylococcus aureus | Right temporal lobe | 9.06 |
| 52 | 80 | male | GBM, IDH wildtype, MGMT methylated | Right frontal lobe | 43.47 |
| 53 | 55 | male | GBM, IDH wildtype, MGMT methylated | Left parietal lobe | 32.33 |
| 54 | 43 | male | GBM, IDH wildtype, MGMT un-methylated | Right temporal lobe, basal ganglia | 69.02 |
| 55 | 81 | male | GBM, IDH wildtype, MGMT methylated | Left frontal lobe | 36.26 |
| 56 | 69 | male | GBM, IDH wildtype, MGMT un-methylated | Right temporal lobe | 128.12 |
| 57 | 67 | male | GBM, IDH wildtype, MGMT un-methylated | Right frontal lobe | 35.91 |
| 58 | 61 | male | GBM, IDH wildtype, MGMT methylated | Left parietal lobe | 36.39 |
| 59 | 78 | male | GBM, IDH wildtype, MGMT un-methylated | Right frontal lobe | 53.11 |
| 60 | 49 | female | GBM, IDH wildtype, MGMT methylated | Left parietal lobe | 74.05 |
| 61 | 69 | male | GBM, IDH wildtype, MGMT un-methylated | Right frontal lobe | 35.41 |
| 62 | 59 | male | GBM, IDH wildtype, MGMT un-methylated | Right temporal lobe | 70.66 |
| 63 | 71 | female | GBM, IDH wildtype, MGMT un-methylated | Right occipital lobe | 80.16 |
| 64 | 73 | female | GBM, IDH wildtype, MGMT methylated | Right occipital lobe | 69.39 |
| 65 | 74 | female | GBM, IDH wildtype, MGMT methylated | Right frontal lobe | 54.39 |
| 66 | 58 | male | GBM, IDH wildtype, MGMT methylated | Right frontal lobe, basal ganglia | 31.61 |
| 67 | 78 | female | GBM, IDH wildtype, MGMT un-methylated | Right temporal lobe | 84.31 |
| 68 | 60 | female | GBM, IDH wildtype, MGMT un-methylated | Left frontal lobe | 46.57 |
| 69 | 74 | male | GBM, IDH wildtype, MGMT un-methylated | Right frontal lobe | 51.33 |
| 70 | 72 | female | GBM, IDH wildtype, MGMT methylated | Left frontal lobe | 17.71 |
| 71 | 68 | male | GBM, IDH wildtype, MGMT un-methylated | Right temporal lobe | 59.22 |
| 72 | 56 | male | GBM, IDH wildtype, MGMT methylated | Right temporal lobe | 7.87 |
| 73 | 60 | female | GBM, IDH wildtype, MGMT methylated | Left temporal lobe | 16.09 |
| 74 | 47 | male | GBM, IDH wildtype, MGMT un-methylated | Right frontal lobe, genu corporis callosi | 171.86 |
| 75 | 58 | female | GBM, IDH wildtype, MGMT methylated | Left parietal lobe | 32.81 |
| 76 | 84 | male | GBM, IDH wildtype, MGMT methylated | Left temporal lobe | 3.72 |
| 77 | 57 | female | GBM, IDH wildtype, MGMT methylated | Right parietal lobe | 94.38 |
| 78 | 68 | male | GBM, IDH wildtype, MGMT methylated | Right frontal lobe | 43.62 |
| 79 | 60 | male | GBM, IDH wildtype, MGMT un-methylated | Left parietal lobe | 111.77 |
| 80 | 52 | male | GBM, IDH wildtype, MGMT un-methylated | Left parietal lobe | 27.89 |
| 81 | 66 | female | GBM, IDH wildtype, MGMT methylated | Left parietal lobe | 12.68 |
| 82 | 62 | female | GBM, IDH wildtype, MGMT un-methylated | Left frontal lobe | 24.85 |
| 83 | 46 | male | GBM, IDH wildtype, MGMT un-methylated | Left frontal lobe | 124.66 |
| 84 | 66 | female | GBM, IDH wildtype, MGMT unknow | Genu corporis callosi | 40.90 |
| 85 | 87 | male | GBM, IDH wildtype, MGMT methylated | Right frontal lobe | 19.28 |
| 86 | 64 | male | GBM, IDH wildtype, MGMT un-methylated | Left frontal lobe | 43.21 |
| 87 | 78 | female | GBM, IDH wildtype, MGMT un-methylated | Left temporal lobe | 84.85 |
| 88 | 68 | male | GBM, IDH wildtype, MGMT un-methylated | Right parietal lobe | 35.95 |
| 89 | 78 | male | GBM, IDH wildtype, MGMT un-methylated | Right parietal lobe | 89.02 |
| 90 | 72 | male | GBM, IDH wildtype, MGMT methylated | Left temporal lobe | 75 |
| 91 | 69 | male | GBM, IDH wildtype, MGMT methylated | Right frontal lobe | 106.64 |
| 92 | 76 | female | GBM, IDH wildtype, MGMT methylated | Left frontal lobe | 16.24 |
| 93 | 56 | male | GBM, IDH wildtype, MGMT un-methylated | Right temporal lobe | 33.32 |
| 94 | 42 | male | GBM, IDH wildtype, MGMT un-methylated | Right parietal lobe | 130.37 |
| 95 | 23 | female | GBM, IDH wildtype, MGMT methylated | Left frontal lobe | 161.28 |
| 96 | 76 | female | GBM, IDH wildtype, MGMT un-methylated | Right frontal lobe | 88.20 |
| 97 | 50 | female | GBM, IDH wildtype, MGMT un-methylated | Left parietal lobe | 6.09 |
| 98 | 62 | female | GBM, IDH wildtype, MGMT un-methylated | Left frontal lobe | 71.13 |
| 99 | 64 | female | GBM, IDH wildtype, MGMT methylated | Left parietal lobe | 52.87 |
| 100 | 78 | male | GBM, IDH wildtype, MGMT un-methylated | Right frontal lobe | 13 |
| 101 | 47 | female | GBM, IDH wildtype, MGMT methylated | Right frontal lobe | 69.46 |
| 102 | 79 | male | GBM, IDH wildtype, MGMT un-methylated | Left temporal lobe | 132.47 |
| 103 | 82 | male | GBM, IDH wildtype, MGMT methylated | Right temporal lobe | 59.70 |
